# Comparative atrial fibrillation risk across Bruton's tyrosine kinase inhibitors in B‐cell malignancies: A nationwide population‐based study

**DOI:** 10.1111/bjh.70634

**Published:** 2026-06-23

**Authors:** Yoann Zelmat, Loic Ysebaert, Paul Gautier, Agnes Sommet, Margaux Lafaurie, Fabien Despas

**Affiliations:** ^1^ Université de Toulouse, CHU de Toulouse, Service de Pharmacologie Toulouse France; ^2^ Institut des Maladies Cardiovasculaires et métabolique (I2MC), UMR 1297, Université de Toulouse, INSERM Toulouse France; ^3^ Université de Toulouse, CHU de Toulouse, Institut Universitaire du Cancer de Toulouse – Oncopôle, Service d'hématologie Toulouse France; ^4^ Centre de Recherche Contre le Cancer (CRCT), UMR1037, INSERM Toulouse France; ^5^ Université de Toulouse, CHU de Toulouse, Service de Cardiologie Toulouse France; ^6^ Centre d'investigation Clinique CIC1436, CHU de Toulouse, Université de Toulouse, INSERM Toulouse France

**Keywords:** atrial fibrillation, B‐cell chronic lymphocytic leukaemia, Bruton's tyrosine kinase, drug‐related side effects and adverse reactions, pharmacoepidemiology

## Abstract

Bruton's tyrosine kinase inhibitors (BTKi) are widely used in B‐cell malignancies. Ibrutinib is associated with atrial fibrillation (AF), but comparative real‐world evidence on AF risk across BTKi remains limited. We conducted a nationwide new‐user population study using the French national health insurance database (2015–2024). Adults initiating ibrutinib, acalabrutinib, zanubrutinib or venetoclax were included, excluding those with prior AF or non‐overlapping BTKi indications. The primary outcome was AF requiring hospitalization. Hazard ratios (HRs) were estimated using Cox models with inverse probability of treatment weighting (IPTW). Among 26 393 patients, median follow‐up ranged from 167 days in the zanubrutinib cohort to 526 days in the ibrutinib cohort. One‐year incidence of hospitalized AF was highest with ibrutinib (2.3%) versus acalabrutinib (0.9%), zanubrutinib (0.7%) and venetoclax (0.5%). Ibrutinib was associated with a significantly higher AF risk versus acalabrutinib (HR 3.03, 95% confidence interval [CI] 1.84–5.00), zanubrutinib (HR 7.58, 95% CI 2.05–28.06) and venetoclax (HR 9.04, 95% CI 5.81–14.04). No significant differences in AF risk were observed between second‐generation BTKi and venetoclax. Ibrutinib was associated with a substantially higher risk of hospitalized AF than second‐generation BTKi, supporting closer cardiac monitoring in ibrutinib‐treated patients.

## Graphical abstract

Real‐world nationwide data in B‐cell malignancies reveal a markedly increased 1‐year incidence of hospitalized atrial fibrillation with ibrutinib compared with second‐generation Bruton's tyrosine kinase inhibitors and venetoclax, arguing for closer cardiac surveillance.

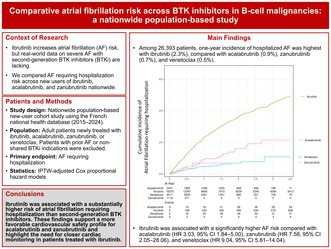

## INTRODUCTION

Bruton's tyrosine kinase inhibitors (BTKi) are oral targeted therapies that are increasingly used as first‐line treatments for chronic lymphocytic leukaemia (CLL) and other B‐cell malignancies.^1^ While atrial fibrillation (AF) is a well‐established adverse drug reaction (ADR) associated with ibrutinib, the AF risk profile of second‐generation BTKi, such as acalabrutinib and zanubrutinib, remains uncertain.^2^, ^3^, ^4^, ^5^

Cardiovascular toxicities, particularly AF, were observed in ibrutinib trials, with subsequent second‐generation BTKi trials suggesting lower rates.^1^, ^4^, ^6^, ^7^ Across randomized studies, reported rates of all‐grade AF ranged from 4 to 17% for ibrutinib, 0 to 9% for acalabrutinib and 2 to 7% for zanubrutinib, while severe AF (according to the Common Terminology Criteria for Adverse Events [CTCAE]) ranged from 3 to 5%, 0 to 5% and 0 to 3% respectively.^1^, ^4^, ^8^, ^9^, ^10^, ^11^ Yet, meta‐analyses of randomized trials failed to demonstrate statistically significant differences in AF risk across BTKi.^3^, ^5^

Importantly, pivotal clinical trials included carefully selected patients with limited cardiovascular comorbidity, whereas real‐world data indicate that about half of ibrutinib users have pre‐existing cardiovascular disease.^12^ This gap between trial populations and clinical practice highlights the need for real‐world comparative evidence. However, such studies remain limited. Although pharmacovigilance studies have consistently reported higher AF rates with ibrutinib compared with second‐generation BTKi,^2^, ^13^, ^14^, ^15^, ^16^ interpreting their results is affected by common pharmacovigilance bias such as underreporting and notoriety bias. Pharmacoepidemiology studies likewise suggest a less favourable cardiovascular safety profile for ibrutinib,^17^, ^18^, ^19^ but lack sufficient detail to capture AF severity or outpatient events and none has evaluated AF risk with zanubrutinib in routine clinical practice.

In this context, we performed a nationwide real‐world comparison of AF risk among new users of ibrutinib, acalabrutinib and zanubrutinib, with a primary focus on AF events requiring hospitalization. Secondary analyses extended these comparisons to all AF events, including outpatient diagnoses and incorporated venetoclax as a non‐BTKi comparator.

## METHODS

### Study design

We conducted a nationwide population‐based cohort study using a new‐user design.

### Data source

This study was conducted using the French national health database (*Système national des données de santé* [SNDS]), which covers >99% of the population (>67 million individuals). The SNDS links pseudonymized individual‐level data, including basic socio‐demographic information (date of birth, sex, insurance scheme and area of residence), hospital discharge summaries, outpatient healthcare reimbursements and mortality data.^20^, ^21^, ^22^, ^23^, ^24^

Hospital data cover both public and private facilities, diagnoses encoded using the International Classification of Diseases, 10th Revision (ICD‐10) and procedures such as cardioversion or atrial flutter ablation. Outpatient claims capture reimbursed healthcare services and drugs dispensed in community pharmacies, coded according to the anatomical therapeutic chemical (ATC) classification. Long‐term disease (LTD) status enables identification of chronic conditions and full reimbursement for related care. Out‐hospital diagnosis and electrocardiography results are not recorded.

### Study population

We identified cohorts of new users of BTKi (ibrutinib, acalabrutinib, zanubrutinib) and venetoclax in the SNDS from 1 January 2015 to 31 December 2024. Venetoclax was used as a non‐BTKi comparator due to overlapping indications with BTKi in B‐cell malignancies and the absence of known AF risk, thereby reflecting baseline AF incidence among treated patients for a B‐cell malignancy.

Eligible patients were aged 18 years or older at the index date, that is the first‐ever dispensation of the study drug. We excluded patients with prior AF, identified through hospitalization records, LTD status or amiodarone dispensation within the preceding 2 years (Table S1, Supplementary Materials).

To ensure clinical comparability across treatment cohorts, we further excluded patients with diagnoses of acute myeloid leukaemia (AML), graft‐versus‐host disease, follicular lymphoma or diffuse large B‐cell lymphoma. The study population was thus restricted to malignancies with shared BTKi indications (CLL, mantle cell lymphoma [MCL], marginal zone lymphoma [MZL], Waldenström macroglobulinaemia [WM]), thereby minimizing heterogeneity related to non‐overlapping indications.

### Exposure

Exposure to ibrutinib, acalabrutinib, zanubrutinib and venetoclax was identified from outpatient dispensing records using ATC codes (Table S1). Based on packaging and usual dispensing patterns, each dispensation was assumed to provide 30 days of treatment. The exposure period extended from the index date until treatment discontinuation, defined as no repeat prescription within 60 days after the last date of dispensation or a switch to another study drug.

### Outcomes

The primary outcome was incident AF requiring hospitalization, defined by an ICD‐10 principal or related diagnosis code of I48 or specific procedures for cardioversion or atrial flutter ablation (Table S1, Supplementary Materials). A secondary analysis applied an extended algorithm that captured both hospitalized and outpatient AF, incorporating LTD status for AF, initiation of amiodarone or initiation of oral anticoagulant therapy in the absence of hospitalization for thromboembolic disease, orthopaedic surgery or valvular disease within the preceding 6 weeks. All‐cause mortality at 1 and 2 years was measured for each cohort.

### Covariates

Risk factors for AF were assessed, including patient demographics (age and sex), treatment indication (CLL, MCL, MZL and WM), prior chemotherapy and baseline comorbidities recorded during the year preceding the index date. Baseline comorbidities included conditions related to the CHA_2_DS_2_‐VASc score (Congestive heart failure, Hypertension, Age ≥75 years [2 points], Diabetes mellitus, Stroke/TIA/thromboembolism [2 points], Vascular disease, Age 65–74 years, Scex category female) and other established risk factors for AF, including hypertension, heart failure, diabetes, prior myocardial infarction and vascular disease (including angina and valvular heart disease) as well as chronic obstructive pulmonary disease (COPD), chronic kidney disease, obesity, obstructive sleep apnoea and thyroid disorders. Covariates were identified using ICD‐10 codes from hospital diagnoses, LTD status and specific drugs or medical devices recorded in the SNDS (Table S1, Supplementary Materials).

### Statistical analyses

Patient baseline characteristics were summarized using means and standard deviations or medians and interquartile ranges for continuous variables, according to their distribution and percentages for categorical variables. Cumulative AF incidence was estimated for each cohort using the Kalbfleisch and Prentice method, accounting for death as a competing risk.

Pairwise comparisons of AF risk among new users of BTKi were performed using Cox‐proportional hazard models to estimate hazard ratios (HRs) and their 95% confidence interval (95% CI). The primary analysis used an as‐treated approach, with follow‐up from the index date until AF, death, treatment discontinuation or switch or study end, whichever occurred first.^25^, ^26^ Proportional hazard assumptions were assessed using visual inspection of log–log survival plots and formally tested with Schoenfeld residuals.^27^

To address confounding, we estimated propensity scores (PS) via logistic regression incorporating all previously listed covariates and treatment initiation year. For each pairwise comparison, these scores reflected the probability of receiving one treatment versus the other.^28^, ^29^ Cox models were then weighted using inverse probability of treatment weighting (IPTW), with weights defined as 1/PS for one cohort and 1/(1‐PS) for the comparator cohort.^30^, ^31^ Post‐weighting balance was confirmed by a standardized difference below 10% and adequate propensity score overlap. E‐values assessed the robustness of findings to unmeasured confounding, representing the minimum association an unmeasured confounder would need with both exposure and outcome to fully explain the observed results.^32^, ^33^

Secondary analyses included a broader AF definition encompassing both hospitalized and outpatient diagnoses, direct comparisons of BTKi versus venetoclax and subgroup analyses restricted to first‐line therapy with CLL without prior chemotherapy. Sensitivity analyses included a 1:1 PS matching, Fine and Gray competing risk models to account for death^34^, ^35^, ^36^ and an intention‐to‐treat (ITT) analysis, in which all patients remained in their initially assigned treatment group until death or the end of follow‐up.^25^, ^26^, ^37^ Associations between baseline characteristics and AF were explored using multivariable logistic regression, with results expressed as odds ratios (ORs) and 95% CIs.

Analyses were performed using SAS Enterprise Guide version 8.3 (SAS Institute Inc., Cary, NC) and R version 4.3.3 (R Foundation for Statistical Computing, Vienna, Austria).

## RESULTS

### Study population

**FIGURE 1:**
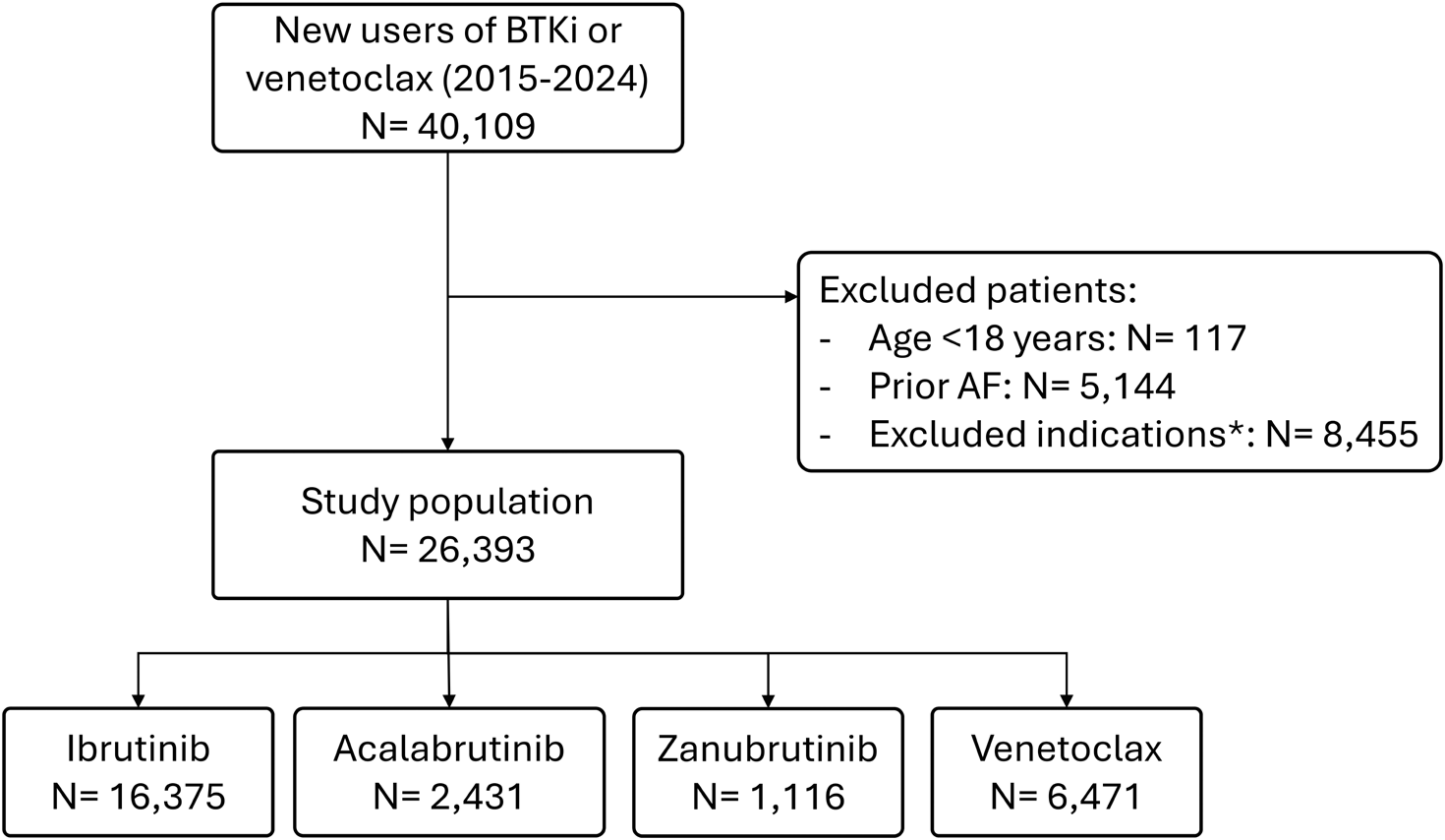
Identification of new users of BTK inhibitors or venetoclax from the French national healthcare claims database. BTK inhibitors, Bruton's tyrosine kinase inhibitors.

Among 40 460 new users of BTKi or venetoclax identified between January 2015 and December 2024, 26 393 new users met the inclusion criteria: 16 375 initiated ibrutinib, 2431 acalabrutinib, 1116 zanubrutinib and 6471 venetoclax (Figure 1, Supplementary Materials). New ibrutinib users peaked around 2021, followed by a progressive shift towards second‐generation BTKi (Figure S1, Supplementary Materials). Baseline characteristics are shown in Table 1. Overall, CLL was the most frequent indication (47.0%), followed by WM (9.0%), MCL (8.3%) and MZL (1.5%). The most common comorbidities were hypertension (57.3%), diabetes (17.8%) and thyroid disorders (12.5%). Patient characteristics varied across treatment cohorts. Median age at treatment initiation ranged from 70 years in the venetoclax cohort to 76 years in the acalabrutinib and zanubrutinib cohorts. New users of ibrutinib had fewer cardiovascular comorbidities (60.2%), while venetoclax initiators had the highest prior chemotherapy exposure (81.8%). New users of zanubrutinib were more frequently treated for WM (23.6%) and MZL (12.2%), whereas acalabrutinib was used predominantly for CLL (70.0%). Median exposure duration was 526 days for ibrutinib, 449 days for acalabrutinib, 167 days for zanubrutinib and 256 days for venetoclax.

**TABLE 1:** Baseline characteristics of patients included in each cohort of new users of BTKi or venetoclax.

Baseline characteristics	Ibrutinib, *N* = 16 375	Acalabrutinib, *N* = 2431	Zanubrutinib, *N* = 1116	Venetoclax, *N* = 6471
Age (years), median (Q1–Q3)	72 [65–79]	76 [70–82]	76 [69–82]	70 [61–76]
Sex				
Men, *n* (%)	10 351 (63.2)	1447 (59.5)	606 (54.3)	4049 (62.6)
Women, *n* (%)	6024 (36.8)	984 (40.5)	510 (45.7)	2422 (37.4)
Indication				
CLL, *n* (%)	7076 (43.2)	1702 (70.0)	277 (25.0)	3355 (52.0)
MCL, *n* (%)	2117 (12.9)	19 (0.8)	29 (2.6)	25 (0.4)
MZL, *n* (%)	218 (1.3)	4 (0.2)	136 (12.2)	27 (0.4)
WM, *n* (%)	2059 (12.6)	26 (1.1)	263 (23.6)	26 (0.4)
Missing, *n* (%)	4905 (30.0)	680 (28.0)	411 (36.8)	3038 (46.9)
Cardiovascular comorbidities, *n* (%)	9865 (60.2)	1588 (65.3)	682 (61.2)	699 (62.6)
Angina pectoris, *n* (%)	580 (3.5)	112 (4.6)	32 (2.9)	278 (4.3)
Hypertension, *n* (%)	9187 (56.1)	1540 (63.3)	667 (59.8)	3734 (57.7)
Heart failure, *n* (%)	1812 (11.1)	181 (7.4)	85 (7.6)	722 (11.2)
History of myocardial infarction, *n* (%)	455 (2.8)	104 (4.3)	39 (3.5)	282 (4.4)
Valvular disease, *n* (%)	1015 (6.2)	143 (5.9)	62 (5.6)	339 (5.2)
Other comorbidities				
COPD, *n* (%)	1463 (8.9)	203 (8.4)	88 (7.9)	474 (7.3)
Chronic kidney disease, *n* (%)	2113 (12.9)	260 (10.7)	108 (9.7)	794 (12.3)
Diabetes, *n* (%)	2775 (16.9)	489 (20.1)	228 (20.4)	1208 (18.7)
Obesity, *n* (%)	2075 (12.7)	325 (13.0)	135 (12.1)	985 (15.2)
Obstructive sleep apnoea, *n* (%)	1014 (6.2)	179 (7.4)	75 (6.7)	443 (6.8)
Thyroid disorder, *n* (%)	1995 (12.2)	350 (14.0)	153 (13.7)	805 (12.4)
History of chemotherapy, *n* (%)	10 744 (65.6)	866 (36.0)	801 (71.7)	5296 (81.8)

Abbreviations: CLL, chronic lymphocytic leukaemia; COPD, chronic obstructive pulmonary disease; MCL, mantle cell lymphoma; MZL, marginal zone B‐cell lymphoma; WM, Waldenström macroglobulinaemia.

### Incidence of AF and all‐cause mortality

**FIGURE 2:**
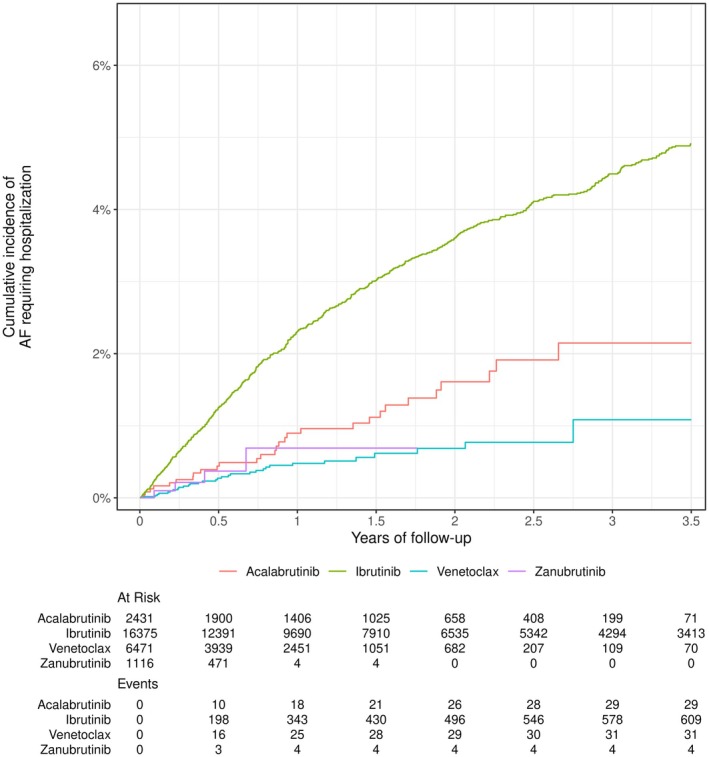
Cumulative incidence of atrial fibrillation requiring hospitalization among new users of BTK inhibitors or venetoclax. AF, atrial fibrillation; BTK inhibitors, Bruton's tyrosine kinase inhibitors.

Ibrutinib was associated with a substantially higher risk of AF requiring hospitalization than second‐generation BTKi. During follow‐up, 740 AF events requiring hospitalization were identified (2.8%), predominantly among ibrutinib new users (*n* = 674, 4.1%) (Figure 2). One‐year cumulative incidence of AF requiring hospitalization was highest in the ibrutinib cohort (2.3%; 95% CI, 2.1–2.6), followed by acalabrutinib (0.9%; 95% CI, 0.6–1.4), zanubrutinib (0.7%; 95% CI, 0.2–1.8) and venetoclax (0.5%; 95% CI, 0.3–0.7). Median time to AF requiring hospitalization onset ranged from 116 days with zanubrutinib to 350 days with ibrutinib. Two‐year incidences ranked similarly: ibrutinib 3.6% (95% CI, 3.3–3.9), acalabrutinib 1.6% (95% CI, 1.1–2.4) and venetoclax 0.7% (95% CI, 0.4–1.0). Using an extended definition of AF that included both hospitalized and outpatient diagnoses, 998 AF events were identified overall (3.8%), most frequently with ibrutinib (5.5%), compared with acalabrutinib (1.6%), zanubrutinib (0.4%) and venetoclax (0.7%). One‐year crude all‐cause mortality was 12.2% (95% CI, 11.7–12.8) with ibrutinib, 6.0% (95% CI, 5–7) in the acalabrutinib cohort, 7.0% (95% CI, 3.7–10.2) with zanubrutinib and 18.4% (95% CI, 17.4–19.5) with venetoclax (Figure S2, Supplementary Materials).

### Comparison of AF risk across BTKi

After IPTW weighting, covariate balance was achieved for all comparisons, with standardized mean differences below 10%. Ibrutinib use was associated with a significantly higher risk of AF requiring hospitalization compared with other BTKi (Table 2): weighted HR (wHR) of 3.03 (95% CI, 1.84–5.00) versus acalabrutinib and 7.58 (95% CI, 2.05–28.06) versus zanubrutinib. The corresponding E‐values were 5.5 (lower 95% CI limit, 3.1) and 14.6 (lower 95% CI limit, 3.5) respectively. No statistically significant differences were observed in AF risk between acalabrutinib and zanubrutinib, nor between second‐generation BTKi and venetoclax.

**TABLE 2:** Comparative risk of atrial fibrillation for ibrutinib versus acalabrutinib, zanubrutinib and venetoclax.

Analyses	Ibrutinib vs. Acalabrutinib wHR (95% CI)	Ibrutinib vs. Zanubrutinib wHR (95% CI)	Ibrutinib vs. Venetoclax wHR (95% CI)
Primary analysis			
AF requiring hospitalization	3.03 (1.84–5.00)	7.58 (2.05–28.06)	9.04 (5.81–14.04)
Secondary analysis			
AF requiring hospitalization and outpatient AF	3.48 (2.27–5.34)	7.63 (2.45–23.76)	9.93 (6.69–14.73)
First‐line treatment for CLL	1.61 (0.91–2.85)	N.A.	30.01 (7.20–125.12)
Sensitivity analyses			
1:1 Matching	3.13 (1.86–5.27)	7.07 (1.85–26.98)	9.47 (5.98–14.97)
ITT analysis	3.29 (2.04–5.30)	7.55 (2.07–27.56)	3.57 (1.49–8.53)
Fine and Gray	2.96 (1.94–4.53)	7.05 (1.97–25.23)	5.21 (3.12–8.69)

Abbreviations: AF, atrial fibrillation; CI, confidence interval; CLL, chronic lymphocytic leukaemia; IPTW, inverse probability of treatment weighting; ITT, intention‐to‐treat; N.A., not applicable; wHR, weighted hazard ratio.

All secondary and sensitivity analyses confirmed these findings (Table 2). Compared to venetoclax, ibrutinib showed a substantially higher risk of inpatient and outpatient AF (wHR 9.04; 95% CI 5.81–14.04), while acalabrutinib (wHR 1.79; 95% CI 0.86–3.74) and zanubrutinib (wHR 1.47; 95% CI 0.34–6.43) showed no significant increase in risk (Figure S3, Supplementary Materials). Across all secondary and sensitivity analyses (Figure S4, Supplementary Materials), ibrutinib consistently showed a higher AF risk versus acalabrutinib (wHR range, 1.6–3.3), zanubrutinib (wHR range, 7.1–7.6) and venetoclax (wHR range, 3.6–30.0).

Several cardiovascular comorbidities, including valvular heart disease (OR 2.40, 95% CI, 1.93–2.95), heart failure (OR 2.28, 95% CI, 1.89–2.74) and hypertension (OR 1.29, 95% CI, 1.06–1.54), were significantly associated with AF requiring hospitalization (Figure S5, Supplementary Materials).

## DISCUSSION

In this nationwide real‐world cohort, ibrutinib was associated with a substantially higher risk of hospitalized AF than acalabrutinib (wHR 3.03), zanubrutinib (wHR 7.58) and venetoclax (wHR 9.04), with no difference observed between second‐generation BTKi and venetoclax. These findings were robust across all secondary and sensitivity analyses.

Baseline characteristics and mortality patterns were consistent with real‐world practice. Shorter follow‐up for zanubrutinib reflected the later market entry of the drug, second‐generation BTKi were preferentially used in older, comorbid patients and indication patterns aligned with approved labels.^38^, ^39^, ^40^ Remarkably, in the present study, the outcome of AF requiring hospitalization closely mirrored severe (grade ≥3 according to CTCAE) AF rates from pivotal clinical trials (ibrutinib 3–5%, acalabrutinib 0–5%, zanubrutinib 0–3%). All‐grade AF incidences were lower than trial‐reported rates (ibrutinib 4–17% vs. 5.5%, acalabrutinib 0–9% vs. 1.6%, zanubrutinib 2–7% vs. 0.4%), reflecting the inherent underestimation of outpatient diagnoses in administrative data. As expected, AF incidence with venetoclax was very low (0.5–0.7%), consistent with the drug's minimal impact on cardiac electrophysiology and confirming venetoclax as a negative comparator reflecting baseline AF risk in this population.^11^

Prior meta‐analyses of randomized trials failed to demonstrate clear AF risk differences across BTKi, likely due to insufficient statistical power, trials heterogeneity and a focus on efficacy over safety.^3^, ^5^ Importantly, pivotal clinical trials carefully included patients without prior cardiac disease. In our real‐world study, we confirmed that more than 60% of patients newly treated with BTKi had pre‐existing cardiovascular comorbidities, which are known to influence the risk of AF and which differed across cohorts. Our study confirms and quantifies the signal of increased hospitalized AF risk with ibrutinib consistently reported by pharmacovigilance analyses, overcoming their inherent limitations.^2^, ^13^, ^14^, ^15^, ^16^

Our findings are consistent with prior real‐world evidence. The OSIRIS study (Observational Study on Ibrutinib Real‐world use In CLL patients in France) reported AF hospitalization in approximately 2.8% of ibrutinib‐treated CLL patients in France.^12^ Two TriNetX‐based observational studies confirmed a higher AF risk with ibrutinib versus acalabrutinib (HR 1.47; absolute excess risk 9%), though of a smaller magnitude, possibly reflecting differences in outcome definitions and database structure.^18^, ^19^

Cardiovascular comorbidities associated with AF in our analysis (valvular heart disease, heart failure and hypertension) align with well‐established risk factors, reinforcing the validity of our findings.^41^

Although the precise mechanisms underlying BTKi‐associated AF remain incompletely understood, evidence suggests that AF is not solely related to BTK inhibition. Ibrutinib inhibits multiple off‐target kinases beyond BTK, including C‐terminal Src kinase (CSK), Tyrosine‐protein kinase Tec (TEC) sarcoma (Src) family kinases, ERBB2/HER2 (Erb‐B2 receptor tyrosine kinase 2/Human epidermal growth factor receptor 2), ERBB4/HER4 (Erb‐B2 receptor tyrosine kinase 4/Human epidermal growth factor receptor 4) and CaMKII (calmodulin‐dependent protein kinase II), which may contribute to atrial remodelling and arrhythmogenesis, an effect not observed with the more selective acalabrutinib and zanubrutinib.^42^, ^43^, ^44^ In vitro studies further suggest that dysregulation of calcium handling and PI3K‐AKT–mTOR signalling (phosphatidylinositol‐3‐kinase‐Ak strain transforming‐mammalian target of rapamycin) may contribute to pro‐arrhythmic effects.^45^, ^46^, ^47^, ^48^ These findings provide only partial biological support for the differential AF risk observed across BTKi, highlighting the need for further translational studies.

### Strengths

This study has several important strengths. First, we leveraged a large nationwide cohort of more than 25 000 patients using a new‐user design with venetoclax as a non‐BTKi comparator not associated with AF risk, offering a pragmatic, though imperfect and estimate of background AF incidence in patients sharing indication with BTKi. This comprehensive database provides near‐complete population coverage, substantial statistical power and limited selection bias. The primary end‐point (AF requiring hospitalization) represents a clinically meaningful and robust outcome, with standardized ascertainment applied uniformly across all cohorts, thereby minimizing differential outcome misclassification.

Although baseline characteristics differed across treatment groups, imbalances were rigorously addressed using IPTW, with robustness confirmed through sensitivity analyses. Competing‐risk analyses using the Fine and Gray model yielded results consistent with the primary analysis, indicating that differential mortality did not materially affect AF risk estimates. Concordant intention‐to‐treat and as‐treated results further suggest minimal impact of treatment discontinuation and attrition bias.

Importantly, the consistency of the associations observed across analyses provides strong evidence for a gradient of AF risk across BTKi. Finally, to our knowledge, this study provides the first large‐scale real‐world pharmacoepidemiological evaluation of AF risk with zanubrutinib in routine clinical practice. It therefore addresses an important evidence gap regarding the cardiovascular safety profile of newer BTKi.

### Limitations

This study has several limitations. First, patients, end‐points and covariates were identified using diagnosis codes from a medico‐administrative database, potentially leading to misclassification. Our definition of severe AF, i.e. as AF requiring hospitalization, likely underestimated the overall incidence. Outpatient AF was captured using an adaptation of the algorithm developed by the French Public Health Agency, based on anticoagulation exposure and the exclusion of alternative indications such as surgery or venous thrombosis.^49^ This approach likely underestimated the true outpatient incidence, though any misclassification would have affected all cohorts similarly, thus not biased comparative estimates and this analysis was considered secondary. Ascertainment of AF events may further vary across institutions due to differing hospitalization thresholds; however, as both BTKi users and comparators were drawn from the same healthcare settings, this bias is likely non‐differential, with limited expected impact on relative risk estimates. Prevalent but undocumented AF at baseline cannot be completely ruled out. Moreover, the indication for BTKi or venetoclax could not be identified for approximately 30% of the study cohort; however, patients with a missing indication were retained in comparative analyses to maximize cohort size. Among them, CLL is the most probable diagnosis, as AML invariably requires hospitalization and would therefore have been identified through inpatient records, supporting the overall clinical homogeneity of the study population after the exclusion of indications not shared with BTKi users.

Residual confounding remains possible, as certain clinical variables were not measurable, including disease severity, line of therapy and lifestyle factors such as body mass index or alcohol use. Anthracycline exposure could not be individually identified in the SNDS, as inpatient chemotherapy agents are not itemized; prior chemotherapy history, ascertained from ICD‐10 codes for chemotherapy‐related hospitalizations, was therefore used as the best available proxy. Still, E‐values for significant IPTW‐weighted HRs ranged from 5.5 to 17.6 (CI limits 3.1–11.1), indicating that unmeasured confounders would need to substantially exceed measured known risk factors (e.g. age, comorbidities) to fully explain the observed differences in AF risk between treatments.^32^ Future studies with access to individual‐level chemotherapy records will be needed to clarify the contribution of anthracycline exposure to AF risk in this population. Temporal changes in treatment practices, patient profiles and cardiovascular monitoring could also have influenced exposure patterns and AF detection, but were mitigated by including, the calendar year of treatment initiation in the propensity score and confirmed by a secondary 1:1 analysis matching on calendar year of initiation, which yielded consistent results. Differential ascertainment of AF related to heightened clinical awareness of ibrutinib‐associated AF over time cannot be fully excluded, though our primary end‐point (AF requiring hospitalization) is primarily driven by clinical severity, limiting the extent of this bias.

Finally, zanubrutinib received French marketing authorization in January 2024, resulting in a shorter median follow‐up (167 days), which likely explains the shorter median time to AF onset (116 days) rather than an inherently earlier occurrence. The precise magnitude of the ibrutinib‐zanubrutinib difference therefore requires longer‐term surveillance and current data preclude a reliable comparison of AF risk between zanubrutinib and acalabrutinib.

### Clinical implications

These findings have important clinical implications for BTKi‐treated patients.^49^ While the relative increase in AF risk with ibrutinib was substantial, the absolute incidence remained modest. This supports the preferential use of second‐generation BTKi or venetoclax in patients with elevated AF risk. Our results underscore the need for cardiovascular assessment before treatment initiation, enhanced rhythm monitoring during therapy with ibrutinib and multidisciplinary collaboration between haematologists and cardiologists. Longer follow‐up is needed to refine AF risk estimates for zanubrutinib and the cardiovascular safety profile of next‐generation BTKi such as pirtobrutinib remains to be evaluated in real‐world settings. These findings complement randomized trial evidence by extending AF risk estimates to a broader, more comorbid real‐world population that remains largely underrepresented in pivotal clinical trials.

## CONCLUSION

In this large, nationwide, real‐world cohort of 26 393 patients treated with BTKi or venetoclax, ibrutinib was associated with a substantially higher risk of AF requiring hospitalization than second‐generation BTKi. These findings support a more favourable cardiovascular safety profile for acalabrutinib and zanubrutinib and provide, to our knowledge, the first large‐scale pharmacoepidemiological evaluation of AF risk with zanubrutinib in routine clinical practice. These results underscore the need for closer cardiac monitoring in patients receiving ibrutinib and may inform treatment selection in patients with elevated risk of AF.

## AUTHOR CONTRIBUTIONS

**Loic Ysebaert:** Conceptualization; methodology; writing – review and editing; validation. **Yoann Zelmat:** Conceptualization; methodology; investigation; validation; software; data curation; visualization; writing – review and editing; writing – original draft. **Fabien Despas:** Supervision; conceptualization; methodology; validation; visualization; writing – review and editing; writing – original draft. **Paul Gautier:** Conceptualization; methodology; validation; writing – review and editing. **Margaux Lafaurie:** Supervision; conceptualization; methodology; visualization; writing – review and editing; writing – original draft; validation. **Agnes Sommet:** Conceptualization; methodology; validation; writing – review and editing.

## FUNDING INFORMATION

This work was conducted within the framework of doctoral training at the University of Toulouse. No dedicated research grant supported this study.

## CONFLICT OF INTEREST STATEMENT

Loic Ysebaert: advisory board member and research grants from AbbVie, AstraZeneca, BeiGene, Bristol Myers Squibb, Gilead/Kite, Johnson and Johnson, Lilly, Roche. All other authors declare no conflicts of interest.

## ETHICS STATEMENT

We conducted the study under the terms of permanent SNDS access granted to hospital researchers according to the French Decree No. 2016‐1871 of 26 December 2016 and French law articles Art. R. 1461‐13 and 14. This study was authorized by the Toulouse University Hospital scientific and ethics committee for the use of data from the SNDS on 18 March 2025 (authorization No. RC31/25/0149). Given that the SNDS is a pseudonymized database, informed consent was not required.

## Supporting information


**Figure S1.** Temporal trend in new users of BTK inhibitors and venetoclax, 2015–2024.
**Figure S2.** Kaplan–Meier curves of overall survival among new users of BTK inhibitors and venetoclax.
**Figure S3.** Cross‐comparison of weighted hazard ratios for atrial fibrillation (AF) requiring hospitalization across BTK inhibitors and venetoclax.
**Figure S4.** Cross‐comparison of weighted IPTW hazard ratios for all atrial fibrillation across BTK inhibitors and venetoclax.
**Figure S5.** Factors associated with atrial fibrillation requiring hospitalization.
**Table S1.** Codes used in the study for the identification of outcomes and comorbidities.

## Data Availability

Individual‐level data from the French National Health Data System (SNDS) cannot be shared by the authors or made directly accessible, in accordance with French legal and regulatory requirements governing health data access and reuse. Researchers may apply for access to SNDS data through the appropriate French national authorities, subject to the required approvals. The study protocol and statistical analysis plan are available from the corresponding author upon reasonable request.
