# Supplementary material for: Comparative atrial fibrillation risk across Bruton's tyrosine kinase inhibitors in B‐cell malignancies: A nationwide population‐based study
Source: Br J Haematol. 2026 Jun 23;209(2):534–43. doi: 10.1111/bjh.70634 (PMC13462094; doi:10.1111/bjh.70634)
Supplement: Supplementary file 1 [file BJH-209-534-s001.docx]

# Supplementary materials

Comparative atrial fibrillation risk across BTK inhibitors in B-cell malignancies: a nationwide population-based study

**Supplementary Figures**

Figure S1. Temporal trend in new users of BTK inhibitors and venetoclax, 2015–2024.

Figure S2. Kaplan-Meier curves of overall survival among new users of BTK inhibitors and venetoclax.

Figure S3. Cross-comparison of weighted hazard ratios for atrial fibrillation requiring hospitalization across BTK inhibitors and venetoclax.

Figure S4. Cross-comparison of weighted IPTW hazard ratios for all atrial fibrillation across BTK inhibitors and venetoclax.

Figure S5. Factors associated with atrial fibrillation requiring hospitalization.

**Supplementary Table**

Table S1. Codes used in the study for the identification of outcomes and comorbidities.

**Figure S1. Temporal trend in new users of BTK inhibitors and venetoclax, 2015–2024.**

Abbreviation: BTK inhibitors: Bruton’s tyrosine kinase inhibitors.

**Figure S2. Kaplan-Meier curves of overall survival among new users of BTK inhibitors and venetoclax.**

Abbreviation: BTK inhibitors: Bruton’s tyrosine kinase inhibitors.

**Figure S3. Cross-comparison of weighted hazard ratios for atrial fibrillation requiring hospitalization across BTK inhibitors and venetoclax.**

Abbreviations: BTK inhibitors: Bruton’s tyrosine kinase inhibitors; CI: confidence interval; IPTW: inverse probability of treatment weighting; HR: hazard ratio.

* indicates statistically significant association

**Figure S4. Cross-comparison of weighted hazard ratios for all atrial fibrillation across BTK inhibitors and venetoclax.**

Abbreviations: BTK inhibitors: Bruton’s tyrosine kinase inhibitors; CI: confidence interval; IPTW: inverse probability of treatment weighting; HR: hazard ratio.

* indicates statistically significant association

**Figure S5: Factors associated with atrial fibrillation requiring hospitalization.**

*Indication: CLL was used as the reference category.

** Age: 18-65 years was used as the reference category

Abbreviations: CLL: chronic lymphocytic leukemia; COPD: chronic obstructive pulmonary disease; MCL: mantle cell lymphoma; MZL: marginal zone B-cell lymphoma; WM: Waldenström macroglobulinemia.

**Table S1. Codes used in the study for the identification of outcomes and comorbidities.**

| Condition | Hospital discharge or long-term disease codes (ICD-10 codes) | Medical acts (CCAM codes) | Drugs (ATC codes) | Medical devices (LPP codes) |
| --- | --- | --- | --- | --- |
| Outcome definition |  |  |  |  |
| Atrial fibrillation | I48 | DEFA002, DENF014, DENF017, DENF018, DENF021, DEPF004, DEPF005, DEPF006, DEPF010, DEPF012, DEPF014, DEPF025, DEPF033 | For outpatient diagnosis: B01AA, B01AE, B01AF, C01BD01 |  |
| Surgery |  | 14.02-14.05 |  |  |
| Veinous thrombosis or thromboembolic disease | I26, I80, I81, I82 | EJQM003, ECQM002, EBQM001, ECQH010, ECQH011, ACQJ001, MZQJ001, GFQL006, GFQL007 |  |  |
| Comorbidities |  |  |  |  |
| Angina pectoris | I20 |  |  |  |
| Chemotherapy | Z511, Z512 |  |  |  |
| Chronic obstructive pulmonary disease | J42, J43, J44 |  |  |  |
| Chronic kidney disease | I120, I131, N032-N037, N052-N057, N18, N19, N250, Z490, Z491, Z492, Z940, Z992 |  |  |  |
| Diabetes | E100, E101, E106, E108, E109, E110, E111, E116, E118, E119, E120, E121, E126, E128-E131, E136, E138-E141, E146, E148, E149 | JVJB001, JVJB002, JVJF002, JVJF003, JVJF004, JVJF005, JVJF006, JVJF007, JVJF008, JVRP004, JVRP007, JVRP008, JVRP007, JVRP008 | A10A, A10B |  |
| Hypertension | I10, I11, I12, I13, I15, I674 |  | C02, C03, C07, C08, C09 |  |
| Heart failure | I50 |  |  |  |
| Myocardial infarction | I21 |  |  |  |
| Obesity | E66 |  |  |  |
| Obstructive sleep apnea syndrome | G473 |  |  | 1132608, 1187880, 1115455, 1192987, 1103446, 1162006, 1124112, 1106663, 1161805, 1112830, 1125301, 1108544, 1127688, 1192148, 1191864, 1122024, 1113545, 1197128, 1196962, 1182598, 1114527, 1124796, 1122337, 1142541, 1115863, 1142417, 1152686, 1179248, 1129888, 1116911, 1154886, 1151250, 1102470, 1119542, 1176340, 1149365, 1141205, 1188661, 1120723, 1103854, 1100637, 1162124, 1185361, 1157459, 1104405, 1189991, 1121131, 1137221, 1159234, 1126275, 1130897, 1126660,1162093, 1166688, 1103156, 1188767, 1124460, 1118904, 1118614, 1133163, 1141197, 1172513, 1148064, 1169586, 1174742, 1104285, 1185496, 1188684, 1113930, 1100040, 1185421 |
| Thyroid disorders | E00-E07 |  | H03AA |  |
| Valvular heart disease | I05, I06, I07, I08, I33, I34, I35, I36, I37, I38, I39 |  |  |  |

Abbreviations: ATC: anatomical therapeutic chemical; CCAM: “Classification Commune des Actes Médicaux” (common classification of medical procedures); ICD-10: international classification of disease; LPP: “Liste des Produits et des Prestations” (list of Products and Services used for medical devices).
